# Reproductive efficiency and shade avoidance plasticity under simulated competition

**DOI:** 10.1002/ece3.2254

**Published:** 2016-06-21

**Authors:** Fatih Fazlioglu, Ali Al‐Namazi, Stephen P. Bonser

**Affiliations:** ^1^ Evolution and Ecology Research Centre School of Biological, Earth and Environmental Sciences UNSW Australia Sydney New South Wales 2052 Australia

**Keywords:** Phenotypic plasticity, plant reproduction, shade avoidance, simulated competition

## Abstract

Plant strategy and life‐history theories make different predictions about reproductive efficiency under competition. While strategy theory suggests under intense competition iteroparous perennial plants delay reproduction and semelparous annuals reproduce quickly, life‐history theory predicts both annual and perennial plants increase resource allocation to reproduction under intense competition. We tested (1) how simulated competition influences reproductive efficiency and competitive ability (CA) of different plant life histories and growth forms; (2) whether life history or growth form is associated with CA; (3) whether shade avoidance plasticity is connected to reproductive efficiency under simulated competition. We examined plastic responses of 11 herbaceous species representing different life histories and growth forms to simulated competition (spectral shade). We found that both annual and perennial plants invested more to reproduction under simulated competition in accordance with life‐history theory predictions. There was no significant difference between competitive abilities of different life histories, but across growth forms, erect species expressed greater CA (in terms of leaf number) than other growth forms. We also found that shade avoidance plasticity can increase the reproductive efficiency by capitalizing on the early life resource acquisition and conversion of these resources into reproduction. Therefore, we suggest that a reassessment of the interpretation of shade avoidance plasticity is necessary by revealing its role in reproduction, not only in competition of plants.

## Introduction

Competition is an important factor controlling plant performance and fitness across environments (Goldberg and Barton 1992; Brown et al. 1998; Weigelt et al. 2005; Bittebiere et al. 2012). However, predictions about how competition shapes reproductive strategies in plants are contradictory. Strategy theory (Grime 1979; Campbell and Grime 1992; Turkington et al. 1993) predicts that under high competition, long‐lived herbaceous plants are predicted to invest in resource uptake (increase in competitive ability, CA) and delay reproduction until reaching some optimum size for reproduction while short‐lived plants favor early intense reproduction. The continuum of early or delayed reproduction strategies has been formalized as r/K selection (MacArthur et al. 1967; Pianka 1970), and these ideas have been fundamental in broader plant strategy theory (see Grime 1979; Taylor et al. 1990; Campbell and Grime 1992; Turkington et al. 1993). From a perspective of life‐history theory, the frequency of disturbances can favor the evolution of short‐lived semelparous species (under high disturbance frequency) and long‐lived iteroparous species (under low disturbance frequency) (see Bell 1976; Bonser 2013). However, intense competition can limit survival and the likelihood of future reproduction of short‐lived and long‐lived species (Ungar 1992; Haag et al. 2004; Tracey and Aarssen 2011). Delaying reproduction under high competition can be hazardous for both semelparous annual and iteroparous perennial plants as they may never reach an optimal size for reproduction (Bonser 2013). Thus, under a life‐history interpretation, both short‐ and long‐lived herbaceous species are predicted to switch to high allocation to reproduction (i.e., increased reproductive efficiency) under increasing competition (Bonser 2013).

Growth form can influence strategies of resource acquisition in plants and, therefore, their competitive abilities. Different plant growth forms can be favored in different habitats. For example, erect growth forms can be favored in low‐light habitats, and rosette or prostrate growth forms can be favored under high‐light habitats (i.e., Bonser and Geber 2005). Therefore, growth form can also affect reproduction through CA and the acquisition of resources that can be allocated to reproduction. The interplay between growth form, CA, and reproduction is fundamental in understanding how plants cope with and evolve in competitive environments. Surprisingly, these relationships between growth form, CA, and reproduction have been infrequently addressed in previous studies (but see Wang et al. 2014 for competition effect on reproductive allocation).

Light is a key limiting resource under competition, and the focus of aboveground competition in plants (Tilman 1988). Plants harvest only a limited range of the light spectrum for photosynthesis (photosynthetically active radiation – PAR), and within this limited range, red wavelength light is preferred to far‐red. This preference causes changes in the ratio of red to far‐red light (R: FR) as light is filtered through or reflected off potentially competing plants (Ballaré et al. 1990; Stuefer and Huber 1998). Variation in light quantity and quality (i.e., decrease in PAR and R: FR ratio) can activate shade avoidance responses in plants (Huber and Wiggerman 1997; Schmitt 1997; Marcuvitz and Turkington 2000; Huber et al. 2004; Weijschedé et al. 2006). Moreover, touching the neighbor leaf tips can also induce shade avoidance responses in plants (de Wit et al. 2012). In a vertical light gradient in dense patches, investment to vertical growth and elongation of internodes would confer greater uptake of light. However, in the horizontal dimension, light availability is unpredictable (Huber 1996). For prostrate species, vertical extension (i.e., better positioning of shaded leaves) is mainly achieved by petiole elongation. However, under homogeneous shade from dominant (tall) neighbors, petiole elongation (associated with light foraging in laterally growing plants) is not beneficial and potentially costly (Weijschedé et al. 2006) as petiole elongation is unlikely to elevate leaves to a better light environment and there are higher construction and maintenance costs in elevating leaves on long petioles. Moreover, depending on environmental conditions, mechanical constraints (i.e., taller and thinner stems cause mechanical failure) may induce selection against the shade avoidance traits (Anten et al. 2009). Therefore, shade avoidance may not always be adaptive.

Shade avoidance is believed to be a mechanism for increasing CA for light when increasing neighbor density limits future and or current light availability. Shade avoidance can allow greater and more efficient resource uptake by investing to light foraging activities such as an increase in petiole length and leaf area or stem elongation (Dudley and Schmitt 1996; Huber and Wiggerman 1997; Smith 2000; Weijschedé et al. 2006). The expression of shade avoidance plasticity can increase performance and fitness of plants across light environments (Ballaré et al. 1990; Huber 1996; Schmitt 1997; Donohue et al. 2000). Because competition for light is highly asymmetric (e.g., Weiner 1990), shade avoidance is consistent with a strategy for increased CA. However, under intense competition, an inferior competitor is not likely to become dominant, and shade avoidance can be a mechanism to maximize resource acquisition to be quickly allocated to reproduction prior to being overtopped by superior competitors. Despite the potential relationship between shade avoidance plasticity and reproduction, few studies have examined shade avoidance plasticity under this life‐history framework. Some previous research points to reduced allocation to reproduction under low‐light quality and quantity (i.e., low R: FR light ratio and photon density) (Brainard et al. 2005; Mahoney and Swanton 2009). Similarly, in a competition experiment, shade from neighbors and mechanical stress (i.e., flexing due to the wind) reduced plant reproduction (Anten et al. 2005). However, no studies have tested alternate predictions of increased CA versus increased reproductive efficiency benefits of shade avoidance.

Competitive ability and plasticity under competition are also predicted to be associated with plant growth form. For example, an erect growth form should have higher CA than a prostrate or rosette growth form (Goldberg and Landa 1991). Multispecies comparisons of erect versus stoloniferous species indicated higher plasticity of erect species in length under shade (Huber 1996; Huber et al. 1998). Also, six stoloniferous species expressed weak responses to changes in R: FR light ratios probably due to poor perception of R: FR (Leeflang 1999). In contrast, a prostrate *Portulaca* species was sensitive to R: FR (Novoplansky et al. 1990). However, shifts in growth form within species are not necessarily associated with a change in shade avoidance plasticity (Bonser and Geber 2005). Thus, it is ambiguous whether there is a consistent relationship between growth forms and plasticity under competition.

We examined the interplay between growth form and life histories in defining competitive and reproductive strategies under spectral shade in herbaceous plants. We conducted a multispecies experiment with 11 species comprising different plant life histories (semelparous annual and iteroparous perennial) and growth forms (erect, ascending, and prostrate). Shade treatments were designed to induce shade avoidance responses associated with adaptive plasticity and CA. Using spectral shade, we excluded the competitive effect of competitors and other aspects of competition (e.g., belowground competition, the density of neighbors) and focused only on the CA of our experimental species. We addressed the following questions: (1) How does competition affect reproductive efficiency and CA of different life histories and growth forms in plants? (2) Is life history or growth form associated with CA? (3) Is shade avoidance plasticity primarily associated with reproductive efficiency under simulated competition?

## Methods

### Study species

We used 11 plant species in this experiment (Table 1). Primulaceae *Anagallis arvensis* (L.) U. Manns & Anderb.*,* Apiaceae *Cyclospermum leptophyllum* (Pers.) Sprague*,* Euphorbiaceae *Euphorbia peplus* L.*,* Brassicaceae *Lepidium africanum* (Burm.f.) DC.*,* Caryophyllaceae *Paronychia brasiliana* DC., Lamiaceae *Stachys arvensis* (L.) L.*,* Asteraceae *Taraxacum officinale* Weber, and Fabaceae *Trifolium dubium* Sibth. are obligate sexual while Plantaginaceae *Cymbalaria muralis* G.Gaertn*., B.Mey. & Schreb.,* Oxalidaceae *Oxalis exilis* A.Cunn. and Fabaceae *Trifolium repens* L. are clonal plants – reproducing both clonally with stolons and sexually. Hereafter, species are referred to their generic names except the two *Trifolium* species – abbreviated as *T. dubium* and *T. repens*. All species are classified as weeds in Australia and widespread through disturbed and stressful areas such as lawns, gardens, farms, pastures, and roadsides (The Atlas of Living Australia – http://www.ala.org.au and PlantNET – The Plant Information Network System of The Royal Botanic Gardens and Domain Trust – http://plantnet.rbgsyd.nsw.gov.au). Seeds were collected from populations along disturbed roadside patches in Sydney, NSW, Australia.

**Table 1:** List of species, growth forms, and life histories of plants included in this experiment

Growth form	Life history	
	Semelparous annual	Iteroparous perennial
Erect	*Cyclospermum leptophyllum*	*Lepidium africanum*
	*Stachys arvensis*	*Taraxacum officinale*
Ascending	*Trifolium dubium*	*Trifolium repens, Oxalis exilis, Cymbalaria muralis*
	*Euphorbia peplus*	
Prostrate	*Anagallis arvensis*	*Paronychia brasiliana*

### Experimental design

In May 2013, seeds of each species were soaked in gibberellic acid (2000 ppm – see Riley 1987) for 3 h to promote germination. Seedlings were transferred to standard plastic pots (16.5 cm radius, 2.4 L volume) filled with growth medium consisting of peat, river sand, nutrients, trace elements, and slow release fertilizer (Osmocote; The Scotts Company, Baulkham Hills, Australia). Plants were grown under two shade treatments: simulated competition (spectral shade) and control. We used a cylinder of green plastic light filter (121 Lee Green; Lee Filters, Andover, UK) (30 cm height × 50 cm diameter) to simulate competition. The filter confers a dense foliage, tropical jungle, or woodland effect and simulates competition by reducing PAR to 33% of daylight and R: FR to 0.2. Light filters were placed inside each pot and held in place by wooden stakes set in the growing medium. Control plants were grown without a filter. Simulated competition and control treatments were replicated eight times per species. In total, the experiment consisted of 176 pots (11 species × 2 treatments × 8 replicates). Germinating many more individuals per species in 11 species at the same time was quite challenging. Therefore, in order to keep the same number of replicates for all species and maximize the number of species (to include a large number of species), we selected eight healthy individuals. Replicates were randomly arranged in eight blocks on benches in UNSW Australia glasshouses (temperature range 20–24°C), and plants were hand‐watered as required (typically three times per week).

### Data collection

The experiment was conducted over 25 weeks from June 2013 to January 2014 (from the beginning of the Austral winter to midsummer). During experiment, plant height, leaf number, flowering time, flower and fruit numbers were recorded biweekly. Size at first flowering was recorded as the number of leaves at the initiation of reproduction. Due to dissimilarity in developmental stages and treatment effect on size, plants were harvested at different time periods whenever they started to senesce (e.g., end of flowering in annuals or leaf death following the first flowering in perennials). Using senescence instead of fixed number of days allowed us a correction for differences in developmental states. At harvest, only aboveground parts were collected including the stolons (if any present). Leaves, stems, flowers, and fruits were separated and stored in paper bags to be dried in an oven. All plant materials were kept in drying oven at 60° for 72 h to a constant mass.

### Data analysis

For each species, we calculated an index of relative reproductive efficiency (RRE). RRE represents the efficiency of biomass allocation from vegetative growth to reproductive output in competition treatments relative to no competition treatments (see – Bonser 2013) and calculated as:

(1) $$\text{RRE}=\log[{\left(F/M\right)}_{c}/{\left(F/M\right)}_{\mathrm{nc}}],$$

where (*F*/*M*)_c_ and (*F*/*M*)_nc_ are the mean fruit numbers relative to total biomass in competition and no competition treatments, respectively, for each species. We used the corrected index of relative competition intensity (CRCI) (Oksanen et al. 2006) to estimate the effect of competition on size:

(2) $$\text{CRCI}=\text{arcsin}[\left(P_{c}-P_{\mathrm{nc}}\right)/\text{Max}\left(P_{c},P_{\mathrm{nc}}\right)],$$

where *P*_c_ is performance under competition (calculated separately for total biomass, leaf number, fruit number, reproductive mass); Max is maximum performance value under competition and no competition. We measured CA, that is, the capacity to maintain size and fitness in the presence of competitors and calculated as: CA = 1 − (CRCI).

We also used a plasticity index (PIv) to quantify shade avoidance responses of plants (Valladares et al. 2000):

(3) $$\text{PIv}=\left[\text{Max}\left(X_{c},X_{\mathrm{nc}}\right)-\text{Min}\left(X_{c},X_{\mathrm{nc}}\right)\right]/\text{Max}\left(X_{c},X_{\mathrm{nc}}\right),$$

where *X*_c_ is trait mean under competition; *X*_nc_ is trait mean under no competition.

Analysis of variance (ANOVAs), sequential *t*‐tests (for binary comparisons), and Tukey's HSD tests were applied using JMP Version 10 (SAS Institute, Cary, NC) to test for differences between simulated competition and control treatments in RRE, CA, plasticity, and traits such as total biomass, height, fruit number, reproductive mass, flowering time, size at first flowering. We tested for significant associations between RRE and competition intensity using linear regression. Regressions were performed for life histories and growth forms separately. Test statistics for the regression across all species were also estimated without grouping results into life history and growth form. Regression models were extended through the origin to account for the expectation that where plants did not experience competition, they should not shift reproductive efficiency. Moreover, regression models through the origin can show any significant associations between reproduction and competition, and reproductive efficiency, CA, and plasticity in shade avoidance traits (PIv) by testing whether data points generally sit above or below a *y*‐value of zero. Therefore, we could detect significant positive or negative shifts in reproductive efficiency with CA, and in reproductive efficiency or CA with shade avoidance plasticity. We calculated shade avoidance plasticity using height/biomass ratio, and height separately, as both are associated with adaptive plasticity under shade.

## Results

**Figure 1:**
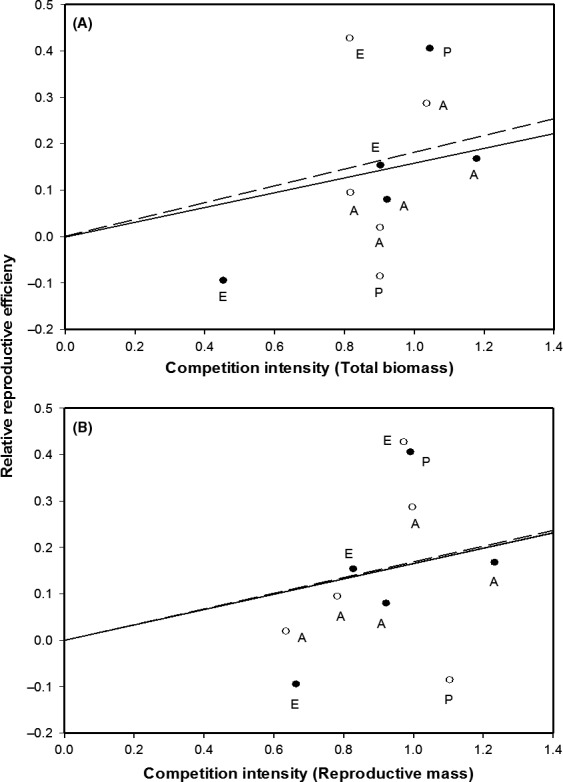
Regression analysis of relative reproductive efficiency versus competition intensity of annual (●, dashed line) and perennial (○, solid line) plants where competition was measured as the loss of total biomass (A) or reproductive mass (B) under simulated competition. A, E, and P labels represent ascending, erect, and prostrate growth forms, respectively.

We found that a significant increase in RRE with increasing competition intensity (*n* = 11, *P* = 0.02 and *n* = 10, *P* < 0.0001 for competition measured as loss of total biomass and loss of reproductive mass, respectively) (Fig. 1). That is, under simulated competition (spectral shade), commitment to reproduction relative to size increased in most species. Within species, fruit number decreased in 10 species and reproductive mass decreased in eight species (one species – *Taraxacum* failed to flower under simulated competition, so ten species were presented) (Fig. 1 and Table 2). Flowering time was delayed under the simulated competition treatment for eight species of 10, and size at first flowering (measured as leaf number) was significantly smaller for 7 of 10 (Table 2). However, *Cyclospermum* and *Stachys* (both annual and erect species) did not start flowering at smaller sizes under simulated competition.

**Table 2:** Trait means (±SE) under control and simulated competition treatments

Species	Total biomass (g)			Height (cm)			Leaf number			Fruit number		
	Control	Treatment	*P*	Control	Treatment	*P*	Control	Treatment	*P*	Control	Treatment	*P*
*Anagallis*	12.9 ± 0.8	1.7 ± 0.3	<0.0001	10.1 ± 0.4	28.4 ± 3.3	<0.0001	1913 ± 79	239 ± 32	<0.0001	157 ± 17	54 ± 9	0.0002
*Cyclospermum*	6.9 ± 0.7	3.9 ± 0.5	0.003	67.6 ± 2.3	85.2 ± 5.7	0.012	840 ± 96	310 ± 21	<0.0001	111 ± 23	50 ± 9	0.031
*Cymbalaria*	10.6 ± 1.3	1.5 ± 0.2	<0.0001	9.6 ± 1	23.6 ± 3.1	0.0007	428 ± 53	98 ± 11	<0.0001	148 ± 13	40 ± 4	<0.0001
*Euphorbia*	4.6 ± 0.3	0.9 ± 0.2	<0.0001	25.5 ± 1.5	34.0 ± 3.5	0.044	1084 ± 55	329 ± 51	<0.0001	328 ± 32	80 ± 18	<0.0001
*Lepidium*	5.0 ± 0.4	1.4 ± 0.1	<0.0001	64.6 ± 1.5	65.6 ± 3.8	0.802	252 ± 27	111 ± 21	<0.001	224 ± 67	162 ± 45	0.450
*Oxalis*	3.9 ± 0.4	1.1 ± 0.2	<0.0001	12.8 ± 0.7	22.3 ± 1.6	<0.0001	120 ± 13	45 ± 6	<0.0001	64 ± 14	22 ± 5	0.011
*Paronychia*	3.0 ± 0.4	0.6 ± 0.4	0.005	7.4 ± 0.7	11.0 ± 3.1	0.242	2480 ± 258	591 ± 258	<0.001	403 ± 98	71 ± 69	0.105
*Stachys*	6.9 ± 0.8	1.5 ± 0.1	<0.0001	19.2 ± 2.5	15.4 ± 0.9	0.186	523 ± 72	510 ± 43	0.88	512 ± 94	156 ± 17	0.002
*Trifolium dubium*	2.7 ± 0.8	0.2 ± 0.05	0.006	5.8 ± 0.8	8.8 ± 2.5	0.271	192 ± 55	20 ± 5	<0.008	107 ± 32	12 ± 3	0.025
*Trifolium repens*	25.2 ± 2.3	5.4 ± 1.6	<0.0001	11.6 ± 0.5	31.7 ± 2.3	<0.0001	504 ± 51	97 ± 14	<0.0001	22 ± 8	5 ± 1	0.047
*Taraxacum*	7.1 ± 0.5	2.6 ± 0.4	<0.0001	15.6 ± 1.1	40 ± 1.4	<0.0001	31 ± 1	17 ± 1	<0.0001	5 ± 1	…	…

Species	Reproductive mass (g)			Flowering time (days)			Size at first flowering (leaf number)		
	Control	Treatment	*P*	Control	Treatment	*P*	Control	Treatment	*P*
*Anagallis*	1.9 ± 0.3	0.3 ± 0.1	0.0003	137 ± 3	151 ± 2	0.001	1766 ± 96	178 ± 21	<0.0001
*Cyclospermum*	1.1 ± 0.2	0.4 ± 0.1	0.006	69 ± 3	105 ± 3	<0.0001	137 ± 47	217 ± 20	0.137
*Cymbalaria*	2.8 ± 0.4	0.4 ± 0.1	<0.0001	61 ± 2	97 ± 4	<0.0001	87 ± 15	27 ± 7	0.003
*Euphorbia*	0.9 ± 0.1	0.2 ± 0.1	<0.0001	60 ± 2	68 ± 5	0.155	595 ± 78	74 ± 11	<0.0001
*Lepidium*	0.4 ± 0.1	0.1 ± 0.03	0.001	69 ± 2	145 ± 8	<0.0001	141 ± 21	47 ± 5	0.0007
*Oxalis*	0.8 ± 0.1	0.2 ± 0.04	0.0004	60 ± 1	85 ± 5	0.0006	42 ± 5	14 ± 1	<0.0001
*Paronychia*	0.3 ± 0.1	0.03 ± 0.02	0.101	125 ± 3	158 ± 4	0.003	1496 ± 497	490 ± 10	0.280
*Stachys*	2.2 ± 0.3	0.6 ± 0.1	<0.0001	53 ± 2	60 ± 1	0.008	102 ± 17	84 ± 14	0.424
*Trifolium dubium*	0.9 ± 0.3	0.05 ± 0.02	0.007	57 ± 4	70 ± 3	0.014	21 ± 5	9 ± 2	0.034
*Trifolium repens*	1.8 ± 0.9	0.7 ± 0.1	0.313	122 ± 14	158 ± 4	0.067	224 ± 51	79 ± 20	0.037
*Taraxacum*	2.4 ± 0.3	…	…	76 ± 6	…	…	23 ± 2	…	…

*P*‐values represent results of one‐factor ANOVAs and *t*‐tests.

**Figure 2:**
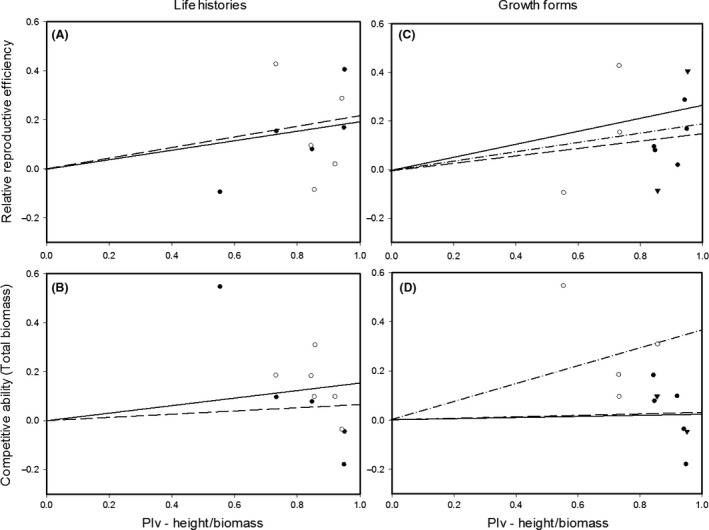
Regression analysis for relative reproductive efficiency and competitive ability versus shade avoidance plasticity (plasticity index**‐** PIv) across life histories and growth forms. A and B represent regression lines for annual (●, dashed line) and perennial (○, solid lines) plants, respectively. C and D represent regression lines for prostrate (▼, solid line), ascending (●, dashed line), and erect (○, dash‐dot line) plants, respectively.

We observed a significant shade avoidance response in most species as spectral shade treatment decreased total biomass and number of leaves in most species, but mean height increased in seven species (Table 2). We found no significant difference between the CA of semelparous annual and iteroparous perennial species (Table 3). However, erect species expressed significantly greater CA (only in terms of leaf number) than ascending and prostrate species (Table 3). Plasticity in height and height‐to‐biomass ratio was associated with an increase in reproduction (Fig. 2 and see also Appendix S1 for analysis of regression). We found a significant association between reproductive efficiency and shade avoidance plasticity (*n* = 10, *P* = 0.02) but no significant association between CA and shade avoidance plasticity (*n* = 11, *P* = 0.14) (Fig. 2). When we group species into erect versus other growth forms, test statistics were nonsignificant for both reproductive efficiency (*n* = 3, *P* = 0.06 for erect; *n* = 7, *P* = 0.32 for other growth forms) and CA (*n* = 4, *P* = 0.10 for erect; *n* = 7, *P* = 0.65 for other growth forms).

**Table 3:** Mean (±SE) competitive ability (CA) for plants across life histories and growth forms

Factors	Life history			Growth form			
	Annual	Perennial	*P*	Ascending	Erect	Prostrate	*P*
	Mean ± SE	Mean ± SE		Mean ± SE	Mean ± SE	Mean ± SE	
CA (total biomass)	0.10 ± 0.12	0.14 ± 0.05	0.75	0.03 ± 0.06	0.28 ± 0.1	0.03 ± 0.07	0.09
CA (leaf number)	0.27 ± 0.19	0.27 ± 0.08	0.98	0.13 ± 0.07	0.56 ± 0.15	0.03 ± 0.1	0.03
CA (fruit number)	0.19 ± 0.09	0.3 ± 0.12	0.65	0.12 ± 0.06	0.46 ± 0.14	0.16 ± 0.12	0.09
CA (reproductive mass)	0.07 ± 0.09	0.10 ± 0.08	0.82	0.09 ± 0.10	0.18 ± 0.09	−0.05 ± 0.06	0.47

Competitive ability was measured for four performance traits: total biomass, leaf number, fruit number, and reproductive mass. *P*‐values represent results of one‐factor ANOVAs and *t*‐tests.

There were no differences between annual and perennial plants in terms of the plasticity of shade avoidance traits. However, across different growth forms, height‐to‐biomass ratios were greater in prostrate and ascending species than erect growth form species (Table 4). The interaction between life history and growth form in CA was significant only when CA was calculated using seed number (Table 5). Overall, spectral shade induced shade avoidance that was associated with an increase in reproductive efficiency, but generally not in CA (with the exception of erect species).

**Table 4:** Mean (±SE) phenotypic plasticity for shade avoidance traits across life histories and growth forms

Factors	Life history			Growth form			
	Annual	Perennial	*P*	Ascending	Erect	Prostrate	*P*
	Mean ± SE	Mean ± SE		Mean ± SE	Mean ± SE	Mean ± SE	
Plasticity index: Height	0.33 ± 0.08	0.43 ± 0.10	0.43	0.45 ± 0.07	0.26 ± 0.13	0.49 ± 0.16	0.34
Plasticity index: Height/biomass	0.81 ± 0.08	0.86 ± 0.03	0.51	0.90 ± 0.02	0.72 ± 0.06	0.90 ± 0.05	0.03

*P*‐values represent results of one‐factor ANOVAs and *t*‐tests.

**Table 5:** Results of a two‐factor ANOVA on the significance of life history and growth form in explaining variation in competitive ability (CA)

Source of variation	df	CA (total biomass)	CA (leaf number)	CA (fruit number)	CA (reproductive mass)
		*P*	*P*	*P*	*P*
Life history	1	0.60	0.79	0.28	0.87
Growth form	2	0.16	0.08	0.03	0.58
Life history × Growth form	2	0.68	0.65	0.11	0.26

Competitive ability was measured from four performance traits: total biomass, leaf number, fruit number, and reproductive mass. *P*‐values represent results of two‐factor ANOVA.

## Discussion

We found that plants expressed higher reproductive efficiency under simulated competition. That is, under competition, species invested more resources to reproduction and initiated reproduction at smaller sizes than for those plants growing in the absence of competition. Therefore, our results support a life‐history theory of reproduction under competition which predicts that short‐lived (annual) plants can express a competitive strategy and intense competition induces a shift to early and efficient reproduction in plants across life histories and growth forms (Bonser and Ladd 2011; Bonser 2013). Allocation of resources to survival or growth rather than reproduction can increase lifetime fitness in the long term (Sih 2004; Nicotra et al. 2010) if there is a prospect for long‐term survival and some growth. In our experiment, spectral shade induced an increase in allocation to reproduction for most species except one annual erect species (*Cyclospermum*) whose growth (i.e., size at first flowering) was not severely affected by the simulated competition treatment, and one perennial prostrate species (*Paronychia*) that did not reach minimum size threshold of reproduction under simulated competition.

All species exhibited highly significant plasticity across competition treatments (Table 2). Decreased F: FR ratio due to the light filter created a competition effect on plants and induced plastic shade avoidance responses (Ballaré et al. 1990; Schmitt 1997; Stuefer and Huber 1998). As a result of simulated competition, total biomass and number of leaves were decreased, average height increased, flowering time was delayed and most of the species started flowering in smaller sizes when compared to control. These results suggested that even the major developmental stages such as the size at reproduction can be highly plastic rather than being conservative (as indicated in other studies – Callahan and Pigliucci 2002; Santos‐Del‐Blanco et al. 2013; Griffith and Watson 2005; Bonser and Aarssen 2009). The later flowering time was likely due to the slow growth rates associated with the spectral shade treatment. As plants invest more into vegetative growth to be competitive (i.e., a traditional view of the shade avoidance response), they may compromise reproductive efficiency. In our study, shade avoidance plasticity is associated with increased reproductive efficiency but not with CA in prostrate and ascending species. Although shade avoidance is generally believed to be a light competition strategy (Huber and Wiggerman 1997; Weijschedé et al. 2006; Bittebiere et al. 2012), our results suggest that shade avoidance is more likely to be a strategy to maximize early life resource acquisition and conversion to reproduction.

We found no difference between the CA of annual and perennial species. This is a somewhat surprising result because under plant strategy theory, annual species typically predicted to be inferior competitors (Mahmoud and Grime 1976; Goldberg and Landa 1991; Campbell and Grime 1992; Fynn et al. 2005). We measured CA under spectral shade that allowed a simulated competition where there is no effect of competing neighbors. Using simulated competition instead of competing plant individuals excluded other aspects of competition (i.e., belowground competition, changes in resources, identity and density of neighbor species) and allowed us to isolate aboveground CA of species in the experiment. The simulated competition treatment affected the size of annual and perennials in a similar way. However, different growth forms (erect, prostrate, and ascending) expressed different competitive abilities under spectral shade. Erect species exhibited greater CA – produced relatively more leaves under simulated competition when compared to other growth forms. While shade avoidance was associated with CA in erect species, erect species also expressed increased reproductive efficiency with increased shade avoidance plasticity. Moreover, erect species were less affected by the presence of the light filter as growing tall yielded better light quality as they were more likely to reach the top of light filter. Thus, we also effectively simulated what should happen if plants could become more competitive than their neighbors.

In theoretical models, life‐history theory suggests that being semelparous or iteroparous depends on trade‐offs between resource allocation to either vegetative growth (survival) or reproductive output (fecundity) (Friedman and Rubin 2015). That is, the schedule of transformation of resources acquired through growth into fitness through offspring production can determine the evolution of reproductive strategies in plants. Semelparity is generally believed to evolve in response to a low probability of surviving to the next reproductive event (Young 1990; Stearns 1992). We suggest major life‐history traits such as the timing of reproduction and number of reproductive events can be fluid in iteroparous perennial species. Where the probability of surviving to the next reproductive event declines under intense competition, iteroparous species will switch to high reproduction perhaps even at the cost of decreased survival. In our study, when we analyzed semelparous annuals versus iteroparous perennials separately, we found no significant relationship between reproductive efficiency and competition intensity. This result was possibly due to reduced power of the analysis (*n* = 5 for semelparous species, and 5 for iteroparous species), but across all species, there was a significant increase in reproductive efficiency under the simulated competition.

We acknowledge that the relatively low number of species representing each growth form and the low replicate number for each species may limit the findings of this study. For example, the presence of two species to represent prostrate growth form is not likely to be sufficient to make generalizations across growth forms. If a greater number of species was included in the study, we could possibly observe more significant differences between the CA of growth forms (not only in terms of leaf number – Table 3) and phenotypic plasticity of shade avoidance traits. However, inclusion of more species would not likely to affect highly nonsignificant results in the CA (and shade avoidance plasticity) between life histories (semelparous annual vs. iteroparous perennial species) because under simulated competition, all plant species (regardless of being annual or perennial) suffered reduced performance and as a result, there was no clear difference between life histories in terms of CA and shade avoidance plasticity. Therefore, these results should be interpreted cautiously when drawing inferences. However, the interplay between reproductive efficiency, CA, and the plasticity of shade avoidance traits across life histories and growth forms has never been addressed in the literature, and our experiment might promote further research about these fundamental questions in future. Despite sample sizes of eight individuals per species and 11 different species, we found a significant increase in reproductive efficiency across species under simulated competition and a significant association between reproductive efficiency and shade avoidance plasticity. These findings would not likely change under an expanded experimental design.

Overall, we demonstrated that shade avoidance plasticity could boost reproductive efficiency by allowing reproduction at smaller sizes. In this sense, the evolution of shade avoidance trait plasticity can enhance plant survival at the individual level by increasing the reproduction rate relative to size at the expense of a reduction in total reproductive output at the population or community level (Weiner 2004). Fluctuations in overall reproduction and fitness at the community level have subtle implications for our agricultural practices, conservation, and restoration management, and our research supports developing ideas on how competition shapes ecological strategies of short‐lived (semelparous annual) and long‐lived (iteroparous perennial) species. Our results should initiate a rethinking of the interpretation of shade avoidance plasticity by shedding light on its possible role in the reproduction of plants rather than as a general adaptation to increase CA.

## Conflict of Interest

None declared.

## Supporting information


**Appendix S1.** Regression analysis for relative reproductive efficiency and competitive ability versus shade avoidance plasticity (Plasticity Index‐Height) across life histories and growth forms.
