# Supplementary material for: Reproductive efficiency and shade avoidance plasticity under simulated competition
Source: Ecol Evol. 2016 Jun 21;6(14):4947–57. doi: 10.1002/ece3.2254 (PMC4979719; doi:10.1002/ece3.2254)
Supplement: Supplementary file 1 — Appendix S1. Regression analysis for relative reproductive efficiency and competitive ability versus shade avoidance plasticity (Plasticity Index‐Height) across life histories and growth forms. [file ECE3-6-4947-s001.docx]

**Appendix S1:** Regression analysis for relative reproductive efficiency and competitive ability versus shade avoidance plasticity (Plasticity Index-Height) across life histories and growth forms. Figures A and B represent regression lines for annual (●, dashed line) and perennial (○, solid line) plants, respectively. Figures C and D represent regression lines for prostrate (▼, solid line), ascending (●, dashed line) and erect (○, dash-dot line) plants, respectively.

Test statistics for regression through origin analysis across all species (without grouping results into life history and growth form) indicated non-significant result for reproductive efficiency versus plasticity (n=10, *p*= 0.06) and non-significant result for competitive ability versus plasticity (n=11, *p*= 0.22). When we group species into erect versus other growth forms, test statistics were significant for reproductive efficiency (n=3, p=0.87 for erect; n=7, p=0.03 for other growth forms) but non-significant for competitive ability (n=4, p=0.17 for erect; n=7, p=0.64 for other growth form.
