# Supplementary figures and images for: Reproductive efficiency and shade avoidance plasticity under simulated competition
Source: Ecol Evol. 2016 Jun 21;6(14):4947–57. doi: 10.1002/ece3.2254 (PMC4979719; doi:10.1002/ece3.2254)

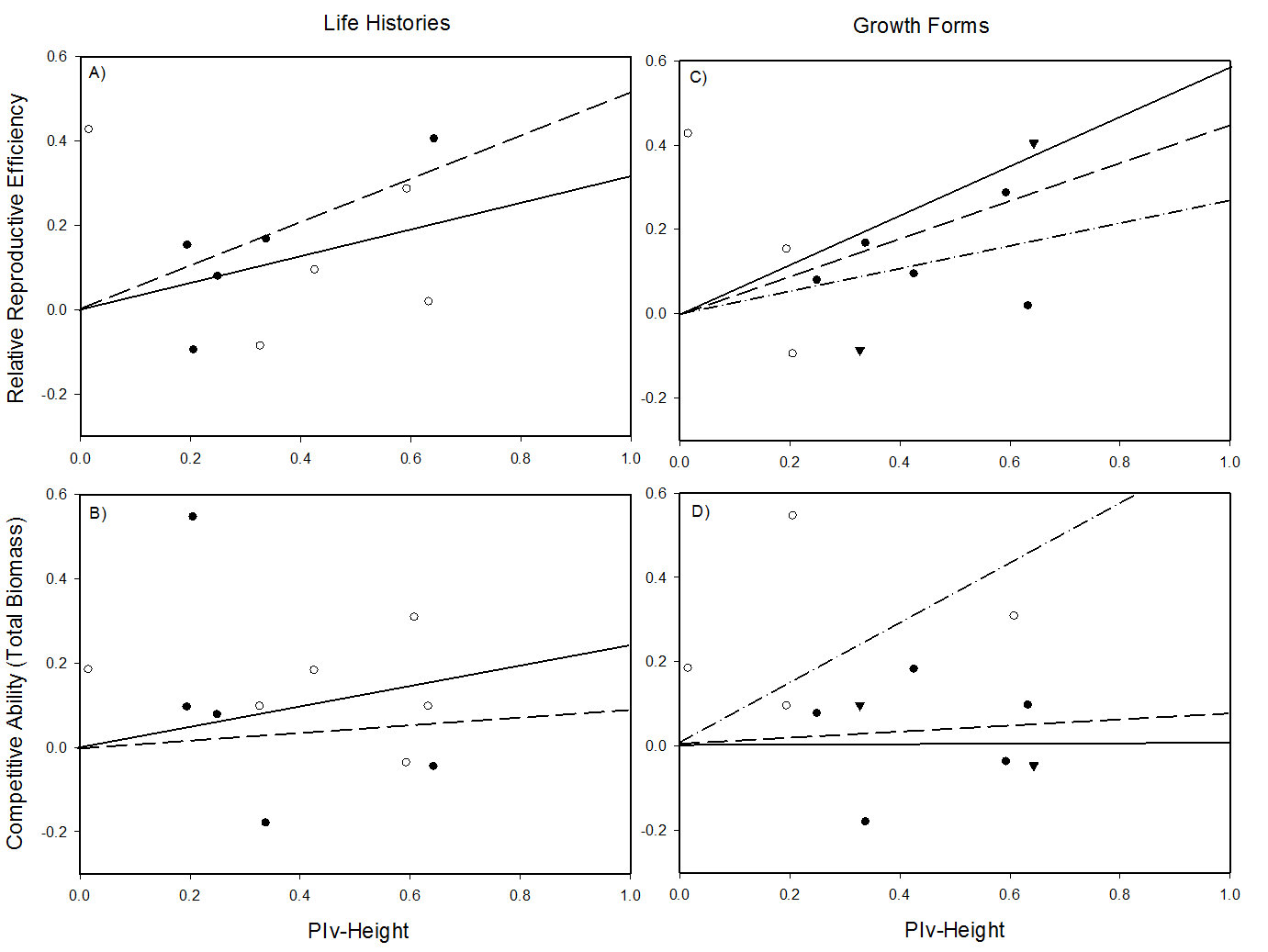

Supplement: Supplementary file 2 [file ECE3-6-4947-s002.tif]
